# Characteristics of balance performance in the Chinese elderly by age and gender

**DOI:** 10.1186/s12877-021-02560-9

**Published:** 2021-10-25

**Authors:** Hongmei Wu, Yifan Wei, Xiangqi Miao, Xia Li, Yang Feng, Zhenzhen Yuan, Peng Zhou, Xiaolei Ye, Jianhong Zhu, Yu Jiang, Qinghua Xia

**Affiliations:** 1 Department of Preventive Medicine, Institute of Nutrition and Diseases, Wenzhou Medical University, Wenzhou, 325035 People’s Republic of China; 2 Department of Traditional Chinese Medicine, Shanghai University of Traditional Chinese Medicine, Shanghai, 201203 People’s Republic of China; 3 School of Medicine, Hunan Normal University, Changsha, 410013 People’s Republic of China; 4 National Center for AIDS/STD Control and Prevention, Chinese Center for Disease Control and Prevention, Beijing, 102206 People’s Republic of China; 5 Changning Center for Disease Control and Prevention, Shanghai, 200051 People’s Republic of China

**Keywords:** Elderly, Balance, Age, Gender, Static balance, Postural stability, Dynamic balance

## Abstract

**Background:**

Population aging has been an emerging public and health concern globally. Balance performance can be applied as an indicator of functional status and a predictor of health outcomes in the elderly. However, reference data of balance performance in the elderly generated from large scale studies have been very limited. In research and geriatric assessment settings, the age and gender specific data on balance performance are indispensable prerequisites for identifying subpopulation with and at risk of impairments and subsequently implementing targeted interventions in clinics and public health to improve their balance performance.

**Methods:**

A total of 1984 elderly subjects aged 60 to 97 years from community settings in urban China were investigated. The balance performances together with 3 individual domains and 16 items were evaluated using the X16 balance testing scale.

**Results:**

In the elderly, with age increases each item, individual domain, and overall balance performance scores decreased gradually. Meanwhile, individual variations of individual domains and overall balance performance were all increased over age. Relative to levels of 60- years, postural stability and overall balance performance decreased significantly since 65 years old, static balance and dynamic balance capacities started to decrease significantly since 70 years old. There was no significant difference in each balance domain and overall balance performance between men and women. Across age groups, portions of individuals able to perform task 4, 8 and 11 successfully were the lowest amongst their corresponding domains static balance, postural stability, and dynamic balance, respectively. Similar patterns were observed in both men and women. Balance performances were categorized into poor, fair, and good groups with scores of 0 to 10, 11 to 17, and 18 to 20, respectively. With increases of age, proportions with poor and fair balance capacities elevated stably.

**Conclusions:**

In the elderly, with advances in age, abilities of overall balance performance, individual domains of static balance, postural stability, and dynamic balance, and successful performances on specific tasks declined gradually and stably. The deterioration started to be obvious since 65–75 years. Men and women had similar patterns.

**Supplementary Information:**

The online version contains supplementary material available at 10.1186/s12877-021-02560-9.

## Background

The pace of population aging around the world has been increasing progressively and rapidly for decades. Global population aged 65 years and over were 151.1 million in 1960, 328.2 million in 1990, and 702.9 million in 2019, accounted for 4.98%, 6.16%, and 9.11%, respectively, indicating a double of the elderly population every 30 years in number and accelerating increase in proportion. Nearly all countries over the world have been experiencing growths in the number and proportion of the elderly in their population. In China, the population aged 65 years and over were 24.4 million in 1960, 66.3 million in 1990, and 164.5 million in 2019, accounted for 3.69%, 5.63%, and 11.47% of the total population, respectively, showing higher increasing rate in contrast to the pace worldwide. Over the upcoming decade, numbers of global and Chinese population aged 65 or over will project to 997.5 and 247.0 million, which will account for 11.67% and 16.87% of their total population, respectively [1].

Population aging is a triumph of development, which is mainly attributable to improved nutrition, sanitation, medical advances, health care, education and economic wellbeing. However, it comes together with challenges to individuals, families, societies and the global community in terms of health status [2]. With increases of age in the elderly, gradual declines in physiological and functional conditions are predictable, consequently their health statuses have visibly deteriorated.

Balance performance has been considered as an indicator of health status in the elderly. The ability of maintaining balance is one imperative component part in carrying out most daily activities independently and successfully [3, 4].

There is no universally accepted definition for human balance, balance herein is described as the ability to maintain various positions and achieve or restore the state of postural equilibrium during activities. For example, the maintenance of specified postures such as sitting or standing, automatic responses to voluntary body or extremity movements such as movements between postures, and reactions to external disturbances [5–7]. Balance can be classified roughly into static balance and dynamic balance. Static balance refers to abilities to maintain a steady position in sitting or standing on a fixed, firm, unmoving base of support. Dynamic balance is abilities to maintain or regain the center of mass within the base of support when the body is moving [5, 8].

A great number of studies showed that balance performance in the elderly declined in contrast to the young adults and middle-aged adults, and among the elderly balance abilities deteriorated with advances in age [9–15]. Previous investigations evaluated balance performance with various approaches, such as functional base of support, functional reach test, the timed up and go test, static balance, etc. However, reference data of balance performance in the elderly by age and gender generated from large scale studies have still been lacking.

Mini-Balance Evaluation Systems Test (Mini-BESTest) offers a unique, brief clinical rating scale for dynamic balance. It helps in directing the specific treatments for the patients and identifying the specific system affected and change with therapy. But it requires equipments and expertised raters, also it needs 10–15 min to administer, and longer depending upon the severity of conditions, which make it may not be practical for regular use in community settings for large scaled screening or evaluation [16].

The Berg Balance Scale (BBS) is the most commonly used assessment tool for stroke rehabilitation in the clinic and has been frequently applied to identify and evaluate balance impairment in the elderly. The scoring system of the BBS is subtle, each item is scored on a scale from 0 to 4, and the differentiation from points 1 to 3 requires careful attention from the investigators. Meanwhile, this scale takes 15 to 20 min to complete. Which makes BBS not suitable for large scaled screening in community settings [6].

The X16 balance testing scale for the elderly was designated for use in community setting and large scale screening. One significant strength of the X16 scale was it was practical and reliable for use in assessments of overall balance performance and individual domains including static balance, postural stability, and dynamic balance simultaneously [17]. In present study a total of 1984 elderly subjects in the unique community context of urban China were investigated and their balance performances were evaluated using the X16 scale. This study established normative data, specified variations between age groups, recognized differences between genders, identified high risk subpopulation at overall balance, individual domain, and specific task levels, and provided guidances on further examination and intervention measures at clinics and public health levels.

## Methods

### Subjects

The project was approved by Institutional Review Board (IRB) of Changning Center for Disease Control and Prevention, Shanghai. The written informed consents were obtained from all of the participants. All protocols for involving humans were in accordance to guidelines of institutional declaration.

The inclusion criteria included, residents lived at home alone or with family in Shanghai for 6 months or longer per year, men and women, aged 60 years or over, functionally independent, physically active, able to ambulate without assistance from others or assistive devices, and able to understand and answer the interview questions. The exclusion criteria included, living in a nursing home, hospitalization, dementia, visual deficits, unable to finish the test for other health reasons.

The subjects were recruited from Changning district of Shanghai, China. From which 8 resident communities were randomly selected, and 375 families were randomly selected from each community. A total of 3000 families were investigated and there were 2312 individuals aged 60 years or above. All of them were assessed for eligibility, of which 168 individuals were incapable of walking independently and unable to complete the balance test, 92 individuals had cognitive function impairments being unable to understand the study, and 68 individuals had incomplete information, thus 328 individuals were excluded. Finally 1984 subjects were included for analysis.

### Data collection

Data were collected through a face-to-face interview by trained investigators. Balance performance was examined on-site using X16 balance testing scale for the elderly [17]. Domain I is static balance, domain II is postural stability, and domain III is dynamic balance. Items are named as domain number followed by item number, for example, I 2 indicates the item 2 which is in domain I, II 7 indicates the item 7 which is in domain II. Items were numbered consecutively through the whole balance testing scale. Each item is scored from 0 to 1 or 2 points. Zero point indicates the impairment, and the 1 or 2 points indicates independence. The scores for the static balance, postural stability, and dynamic balance domains are ranged from 0 to 4, 0 to 8, and 0 to 8 points, respectively. The total score for balance performance is ranged from 0 to 20 points (Table S1).

### Statistical analyses

EpiData 3.0 (The EpiData Association, Odense, Denmark) was used for data entry, and SPSS 22.0 (SPSS Inc. Chicago, IL, USA) was applied for data processing and statistical analysis.

The data of balance performance scores were expressed as mean ± standard deviation (SD), differences between age groups were analyzed with one-way analysis of variance (ANOVA) followed by Tamhane’s T2 for multiple comparisons, differences between men and women were compared with Student’s *t* test. The categorical data between age groups or between items were analyzed with Chi-squared test followed by Bonferroni correction for multiple comparisons. Balance performances were categorized into groups with Two-Step Cluster Analysis. The significance level was set at 0.05.

## Results

### Age and gender compositions of the elderly

A total of 1984 participants were recruited in this study, the mean age was 70.5 ± 7.5 (mean ± SD) years, and the median age (25th percentile, 75th percentile) was 69.0 (64.0, 76.0) years with ranges from 60 to 97 years. Among the 1984 participants, there were 940 men (47.4%) and 1044 women (52.6%), overall sex ratio (Men/Women) was 0.90, age specific sex ratios were ranged from 0.66 to 1.15 (Table S2).

In general, static balance, postural stability, dynamic balance, and overall balance performance scores decreased gradually with age increases (all *P* < 0.0001). Relative to levels of 60- years, postural stability and overall balance performance decreased to significant levels since 65 years old, static balance and dynamic balance capacities started to decrease significantly since 70 years old (Table 1). In the elderly, individual variations of domains static balance, postural stability, and dynamic balance together with balance performance were all increased gradually over age, and roughly coefficients of variations increased substantially started from 75 years old. Throughout varying age groups, static balance and dynamic balance showed larger variations than postural stability. At 85–97 years, coefficients of variations for static balance and dynamic balance were 61.4% and 66.2%, respectively, while that for postural stability was 49.0% (Table 1).

**Table 1:** Balance performances and variations in the elderly by age

Age (yrs)	n	Static balance		Postural stability		Dynamic balance		Balance	
		Mean ± SD	CV (%)	Mean ± SD	CV (%)	Mean ± SD	CV (%)	Mean ± SD	CV (%)
60-	511	3.76 ± 0.76^a^	20.1	7.70 ± 0.90^a^	11.7	7.73 ± 1.06^a^	13.7	19.20 ± 2.13^a^	11.1
65-	530	3.67 ± 0.87^ab^	23.7	7.42 ± 1.25^b^	16.8	7.54 ± 1.32^ab^	17.5	18.63 ± 2.73^b^	14.7
70-	378	3.54 ± 0.94^b^	26.7	7.28 ± 1.36^b^	18.6	7.46 ± 1.47^b^	19.7	18.27 ± 3.09^b^	16.9
75-	297	3.24 ± 1.21^c^	37.2	6.67 ± 1.86^c^	27.9	6.85 ± 2.24^c^	32.8	16.76 ± 4.49^c^	26.8
80-	165	2.93 ± 1.41^cd^	48.2	6.22 ± 2.02^c^	32.5	6.09 ± 2.85^d^	46.7	15.24 ± 5.39^d^	35.3
85–97	103	2.44 ± 1.50^d^	61.4	5.11 ± 2.50^d^	49.0	4.91 ± 3.25^e^	66.2	12.46 ± 6.57^e^	52.7
Total	1984	3.48 ± 1.07	30.7	7.13 ± 1.61	22.5	7.21 ± 1.91	26.4	17.83 ± 3.94	22.1
F		47.38		77.95		64.38		91.78	
*P*		<0.0001		<0.0001		<0.0001		<0.0001	

Superscript lowercase letters (a, b, c, etc) indicated multiple comparison results among various age groups. Same letters indicated non-significant difference, different letters indicated significant differences in statistics. Significance level was 0.05. Coefficients of variation (CV) was in percent

There was no significant difference in each balance domain and overall balance performance between men and women. However, at specific age groups, there were slight differences in balance capacities and descending trends between men and women. At 70- years, static balance and postural stability scores in men were significantly higher than women (*P* < 0.05 and *P* < 0.01, respectively), balance performance score in men was marginally significantly higher than women (*P* = 0.067). In men, static and dynamic balance scores started to decrease significantly since 75 years old, whereas postural stability and overall balance performance started to decrease since 65 years. In women, dynamic scores started to decrease significantly since 75 years old, while static balance, postural stability, and overall balance performance started to significantly decrease since 70 years (Table 2).

**Table 2:** Balance performance in the elderly by age and gender

Age (yrs)	n	Men				n	Women			
		Static balance	Postural stability	Dynamic balance	Balance		Static balance	Postural stability	Dynamic balance	Balance
60-	228	3.79 ± 0.76^a^	7.78 ± 0.76^a^	7.78 ± 0.96^a^	19.34 ± 1.78^a^	283	3.75 ± 0.76^a^	7.64 ± 1.01^a^	7.70 ± 1.13^a^	19.08 ± 2.37^a^
65-	246	3.69 ± 0.82^a^	7.48 ± 1.24^b^	7.50 ± 1.45^a^	18.67 ± 2.93^b^	284	3.65 ± 0.91^ab^	7.37 ± 1.26^ab^	7.57 ± 1.19^a^	18.59 ± 2.56^ab^
70-	184	3.64 ± 0.81^a^	7.46 ± 1.14^b^	7.47 ± 1.44^a^	18.57 ± 2.81^b^	194	3.44 ± 1.05^bc*^	7.10 ± 1.52^bc*^	7.44 ± 1.50^ab^	17.99 ± 3.31^bc^
75-	159	3.26 ± 1.20^b^	6.63 ± 1.84^c^	6.81 ± 2.32^b^	16.70 ± 4.56^c^	138	3.21 ± 1.22^cd^	6.71 ± 1.88^cd^	6.90 ± 2.16^bc^	16.82 ± 4.43^cd^
80-	82	2.98 ± 1.44^b^	6.12 ± 2.12^cd^	6.10 ± 2.93^b^	15.20 ± 5.58^cd^	83	2.88 ± 1.39^de^	6.31 ± 1.93^d^	6.08 ± 2.78^cd^	15.28 ± 5.22^d^
85–97	41	2.54 ± 1.47^b^	5.27 ± 2.25^d^	5.27 ± 3.01^b^	13.07 ± 5.58^d^	62	2.37 ± 1.53^e^	5.00 ± 2.67^e^	4.68 ± 3.41^d^	12.05 ± 7.16^e^
Total	940	3.52 ± 1.03	7.19 ± 1.55	7.23 ± 1.91	17.94 ± 3.83	1044	3.44 ± 1.10	7.08 ± 1.65	7.20 ± 1.91	17.73 ± 4.03
F		20.84	41.28	24.06	41.08		26.79	38.66	41.29	51.21
*P*		<0.0001	<0.0001	<0.0001	<0.0001		<0.0001	<0.0001	<0.0001	<0.0001

Superscript lowercase letters (a, b, c, etc) indicated multiple comparison results among various age groups. Same letters indicated non-significant difference, different letters indicated significant differences in statistics. Asterisk (*) indicated significant differences between men and women. Significance level was 0.05

**Fig. 1:**
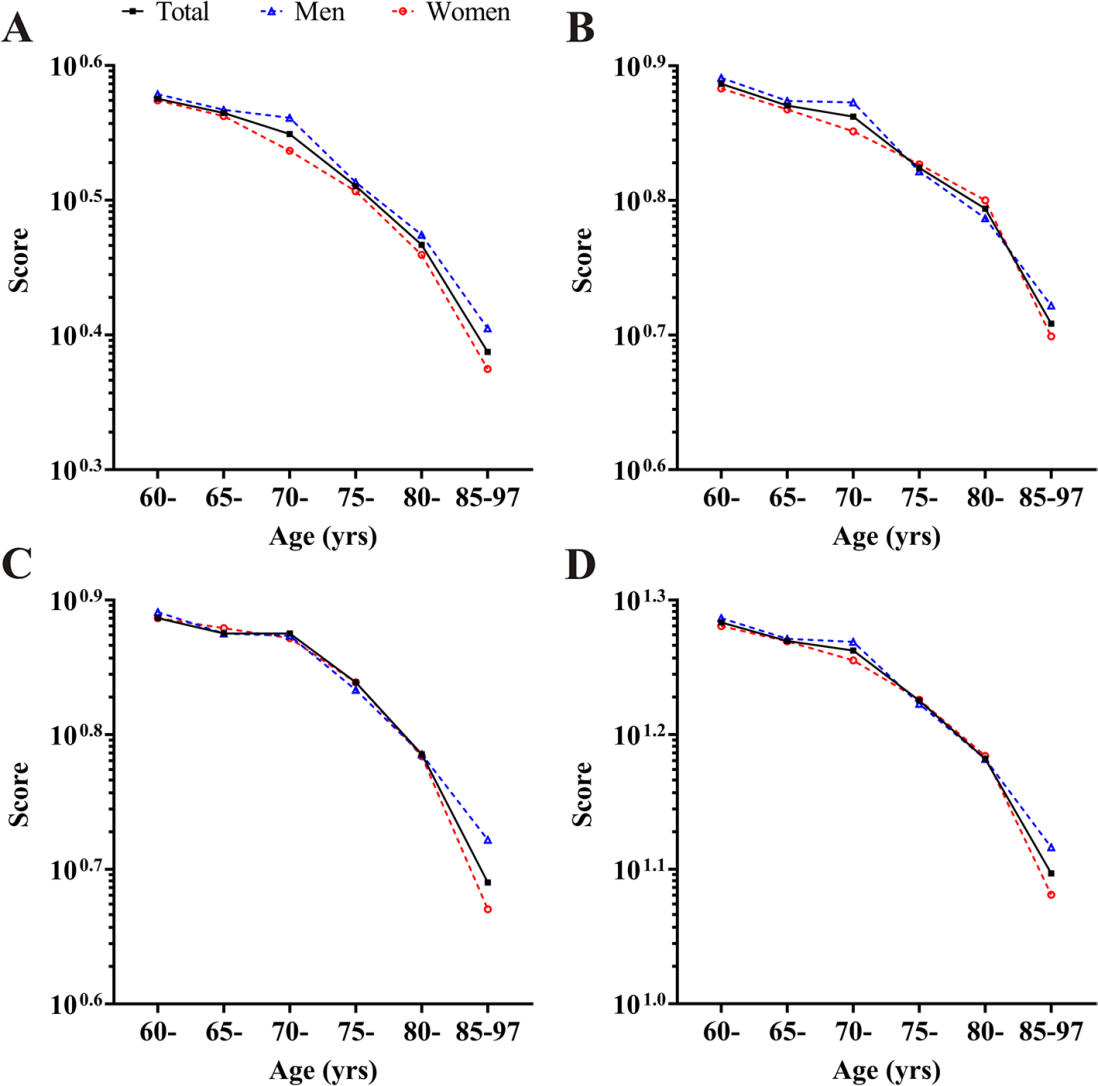
Decline rates of balance performances in the elderly by age. **A** Static balance. **B** Postural stability. **C** Dynamic balance. **D** Balance performance

In terms of descending rate, each balance domain and overall balance performance showed similar patterns. Overall, declines in balance performances were getting faster and faster with increases of age. Before 75 years old, declines were relatively slow, during 70 to 85 years declines were approximately linear, and after 85 years old, declines were the steepest. There were slight differences in decline pattern between men and women. In general, static balance in men was better than women across all ages. During 65 years to 75 years, in men static balance, postural stability, and dynamic balance all maintained at relatively stable level, however in women the decreases in static balance and postural stability were observable. Since 80 years old, declines in each balance domain and overall balance performance in men were less than women, and after 85 years old each balance domain and overall balance performance in men were better than women (Fig. 1).

Tasks in static balance was evaluated. Before 75 years old, over 90% individuals were able to perform tasks 1, 2, and 3 successfully. After 75 years old, performances for tasks 1, 2, and 3 started to decrease gradually. Across all age groups, portions of individuals able to perform task 4 successfully were significantly lower than portions for tasks 1, 2, and 3. For task 4, in 60- years 88.8% individuals were able to perform successfully, in 70- years and 80- years the portions were 75.9% and 55.8%, respectively. And the portion was down to 35.9% in individuals aged 85 years and over whereas the portions were 60.2% to 75.7% for tasks 1, 2, and 3 (Table 3). Men and women had similar patterns (Table S3).

**Table 3:** Static balance in the elderly by age

Age (yrs)	n	I 1		I 2		I 3		I 4	
		n	%	n	%	n	%	n	%
60-	511	490	95.9^a^_A_	492	96.3^a^_A_	487	95.3^a^_A_	454	88.8^a^_B_
65-	530	502	94.7^ab^_A_	500	94.3^a^_A_	498	94.0^a^_A_	446	84.2^a^_B_
70-	378	352	93.1^ab^_A_	356	94.2^ab^_A_	342	90.5^ab^_A_	287	75.9^b^_B_
75-	297	266	89.6^bc^_A_	261	87.9^bc^_A_	248	83.5^bc^_A_	187	63.0^c^_B_
80-	165	138	83.6^cd^_A_	132	80.0^cd^_A_	121	73.3^cd^_A_	92	55.8^c^_B_
85–97	103	78	75.7^d^_A_	74	71.8^d^_A_	62	60.2^d^_A_	37	35.9^d^_B_
Total	1984	1826	92.0 _A_	1815	91.5 _A_	1758	88.6 _B_	1503	75.8 _C_
χ^2^		71.9		108.0		167.3		219.4	
*P*		<0.0001		<0.0001		<0.0001		<0.0001	

Superscript lowercase letters (a, b, c, etc) indicated multiple comparison results among various age groups. Subscript capital letters (A, B, C, etc) indicated multiple comparison results between items. Same letters indicated non-significant difference, different letters indicated significant differences in statistics. Significance level was 0.05

For tasks 5 and 6 in postural stability, there was no significant decrease in performance from 60 to 75 years old, over 90% individuals were able to perform successfully cross these 3 age groups. In 75- years, the portions substantially decreased to 78.8% and 74.7%, respectively. And after 85 years, the portions were down to 49.5% and 42.7%, respectively. In general performances for task 5 and task 6 in the elderly were significantly better than tasks 7 and 8. Success portions for tasks 5 and 6 were 87.0% and 84.7%, respectively, while success portions for tasks 7 and 8 were 76.1% and 72.8%, respectively. Before 85 years old portions of successful performances for tasks 5 to 6 were significantly higher than portions for tasks 7 to 8. There was no significant difference in portions between items over 85 years old (Table 4). Men and women had similar patterns (Table S4 and S5).

**Table 4:** Postural stability in the elderly by age

Age (yrs)	n			II 5				II 6								II 7				II 8					
		2		1		0		2		1		0		2		1		0		2		1		0	
		n	%	n	%	n	%	n	%	n	%	n	%	n	%	n	%	n	%	n	%	n	%	n	%
60-	511	492	96.3^a^_A_	19	3.7	0	0.0	483	94.5^a^_AB_	28	5.5	0	0.0	468	91.6^a^_B_	42	8.2	1	0.2	450	88.1^a^_B_	60	11.7	1	0.2
65-	530	492	92.8^a^_A_	36	6.8	2	0.4	480	90.6^a^_A_	48	9.1	2	0.4	437	82.5^b^_B_	88	16.6	5	0.9	419	79.1^b^_B_	104	19.6	7	1.3
70-	378	350	92.6^a^_A_	26	6.9	2	0.5	342	90.5^a^_A_	34	9.0	2	0.5	293	77.5^b^_B_	80	21.2	5	1.3	274	72.5^b^_B_	93	24.6	11	2.9
75-	297	234	78.8^b^_A_	60	20.2	3	1.0	222	74.7^b^_A_	71	23.9	4	1.3	189	63.6^c^_B_	96	32.3	12	4.0	180	60.6^c^_B_	103	34.7	14	4.7
80-	165	108	65.5^c^_AB_	54	32.7	3	1.8	110	66.7^b^_A_	53	32.1	2	1.2	84	50.9^cd^_C_	74	44.8	7	4.2	85	51.5^cd^_BC_	71	43.0	9	5.5
85–97	103	51	49.5^c^	43	41.7	9	8.7	44	42.7^c^	49	47.6	10	9.7	39	37.9^d^	47	45.6	17	16.5	36	35.0^d^	47	45.6	20	19.4
Total	1984	1727	87.0 _A_	238	12.0	19	1.0	1681	84.7 _A_	283	14.3	20	1.0	1510	76.1 _B_	427	21.5	47	2.4	1444	72.8 _B_	478	24.1	62	3.1
χ^2^		279.6						266.4						245.3						205.1					
*P*		<0.0001						<0.0001						<0.0001						<0.0001					

Superscript lowercase letters (a, b, c, etc) indicated multiple comparison results among various age groups. Subscript capital letters (A, B, C, etc) indicated multiple comparison results between items. Same letters indicated non-significant difference, different letters indicated significant differences in statistics. Significance level was 0.05

Among 8 tasks in dynamic balance, before 75 years old, over 90% individuals were able to perform tasks 9, 10, 12, 13, 14, 15, and 16 successfully. At 80- years old, more than 80% elderly were able to perform tasks 14 and 15 successfully. Performances for task 11 was the worst, since 65 years old portions with success performance were below 90%, till over 85 years old the portion was down to 49.5% while portions for other tasks were ranged from 56.3% to 72.8% (Table 5). Men and women had similar trends across ages (Table S6 and S7).

**Table 5:** Dynamic balance in the elderly by age

Age (yrs)	n	III 9		III 10		III 11		III 12		III 13		III 14		III 15		III 16	
		n	%	n	%	n	%	n	%	n	%	n	%	n	%	n	%
60-	511	498	97.5^a^_A_	494	96.7^a^_A_	470	92.0^a^_B_	495	96.9^a^_A_	496	97.1^a^_A_	502	98.2^a^_A_	501	98.0^a^_A_	496	97.1^a^_A_
65-	530	506	95.5^a^_A_	492	92.8^a^_AB_	468	88.3^a^_B_	502	94.7^a^_A_	501	94.5^a^_A_	513	96.8^a^_A_	507	95.7^ab^_A_	507	95.7^ab^_A_
70-	378	358	94.7^a^_A_	349	92.3^a^_AB_	331	87.6^ab^_B_	350	92.6^ab^_AB_	353	93.4^a^_AB_	366	96.8^a^_A_	364	96.3^a^_A_	348	92.1^b^_AB_
75-	297	258	86.9^b^_ABCD_	249	83.8^b^_ABCD_	237	79.8^b^_CD_	257	86.5^bc^_ABCD_	244	82.2^b^_BD_	270	90.9^b^_A_	269	90.6^b^_AB_	251	84.5^c^_ABCD_
80-	165	130	78.8^bc^_AB_	126	76.4^b^_AB_	108	65.5^c^_B_	126	76.4^cd^_AB_	120	72.7^bc^_AB_	139	84.2^bc^_A_	132	80.0^c^_AB_	124	75.2^cd^_AB_
85–97	103	67	65.0^c^_AB_	59	57.3^c^_AB_	51	49.5^c^_B_	64	62.1^d^_AB_	58	56.3^c^_AB_	75	72.8^c^_A_	71	68.9^c^_AB_	61	59.2^d^_AB_
Total	1984	1817	91.6 _ABC_	1769	89.2 _C_	1665	83.9 _D_	1794	90.4 _BC_	1772	89.3 _C_	1865	94.0 _A_	1844	92.9 _AB_	1787	90.1 _C_
χ^2^		175.7		186.2		171.6		175.9		234.9		143.8		167.9		209.1	
*P*		<0.0001		<0.0001		<0.0001		<0.0001		<0.0001		<0.0001		<0.0001		<0.0001	

Superscript lowercase letters (a, b, c, etc) indicated multiple comparison results among various age groups. Subscript capital letters (A, B, C, etc) indicated multiple comparison results between items. Same letters indicated non-significant difference, different letters indicated significant differences in statistics. Significance level was 0.05

A two-step cluster analysis was applied to identify group segmentations, balance performances were categorized into 3 groups with scores of 0 to 10, 11 to 17, and 18 to 20, which were arbitrarily named poor, fair, and good, respectively. With increases of age, proportions with poor and fair balance capacities elevated gradually, and after 85 years old reached up to 36.9% and 33.0%, respectively (Table 6). There was slight difference in increasing pattern between men and women. In men the proportion with poor balance capacity increased from 12.6% at 75- years old to 29.3% at 85–97 years old, while in women the portion elevated substantially from 8.0% to 41.9% during corresponding age stages (Table S8).

**Table 6:** Categories of balance performance in the elderly by age

Age (yrs)	n	Poor (0–10)		Fair (11–17)		Good (18–20)	
		n	%	n	%	n	%
60-	511	8	1.6^a^	42	8.2^a^	461	90.2^a^
65-	530	13	2.5^a^	84	15.8^b^	433	81.7^b^
70-	378	18	4.8^ab^	66	17.5^bc^	294	77.8^b^
75-	297	31	10.4^b^	82	27.6^d^	184	62.0^c^
80-	165	37	22.4^c^	47	28.5^cd^	81	49.1^c^
85–97	103	38	36.9^c^	34	33.0^d^	31	30.1^d^
Total	1984	145	7.3	355	17.9	1484	74.8

A two-step cluster analysis was applied to identify group segmentations. Superscript lowercase letters (a, b, c, etc) indicated multiple comparison results among various age groups. Same letters indicated non-significant difference, different letters indicated significant differences in statistics. Significance level was 0.05

## Discussion

The present study provided detailed reference information on balance performances in community elderly population. Results demonstrated the subjects with older ages had lower scores for overall balance performance, separate domains, and specific tasks. With increases of age, the proportions of the elderly with impaired balance increased stably and substantially.

Based on these age and gender specific data information on balance performance resulted from this study, in research and geriatric assessment settings, subpopulation and high risk groups with and at risk of specific domain or item impairments at certain age and gender would be able to be identified, then further examinations followed by targeted interventions would be possible and feasible in clinics and public health to improve their balance performance.

In the present study sex ratio of the elderly population aged 60 years and above was within ranges of ratios in China and worldwide, the sex ratio aged 85 years and over was also comparable to the global ratio. In contrast to overall sex ratio of elderly aged 60 years and over, sex ratio of the elderly aged 85 years and above was obviously lower, this pattern was in agreement with data in China and worldwide as well. Women have longer life expectancies than men in world, China, and Shanghai, which is attributable to lower men to women sex ratio in the oldest elderly at least in part [2, 18–21].

To elucidate characters of balance performance with changes of age, subjects were classified into specific age groups with a 5-year interval. There were relatively limited individuals aged 85 years or over, therefore they were combined within 1 group, making a total of 6 age groups for analytic comparisons. The 16 items of X16 balance testing scale were clustered into 3 domains of static balance, postural stability, and dynamic balance [17]. Thus balance performances in the elderly were elucidated in details at levels of total balance performance, separate domains, and individual items.

Elucidations of balance performance stratified by age and gender provided valuable evidence for references and interventions. In the elderly static balance, postural stability, dynamic balance, and overall balance performance were compared between age groups. As expected, in general balance scores decreased gradually with age increase. However, relative to declines in static balance and dynamic balance starting from 70 years old, postural stability started to worsen since 65 years, making overall balance performance started to decrease at 65 years. After 80 years old, both overall balance performance and individual domains started to deteriorate substantially in both men and women. These findings provided of value scientific information for guidance on timely and well focused screenings, examinations, and interventions accordingly. The signature benefits may be aimed policy programs on subpopulations with certain levels of functioning, which would allow for a significantly reduced sample size.

In this study there was no significant difference observed in static balance, postural stability, dynamic balance, or overall balance performance between men and women. Yet in the elderly aged 85 years and over, men had somewhat slightly higher scores in static balance, postural stability, dynamic balance, and overall balance performance than women. Previous studies showed inconsistent results. For example, a report from community dwelling elderly individuals in Brazil found there was no significant difference in scores on Berg Balance Scale regarding gender [22]. While a study based on a Turkish sample showed men had significantly better balance performance in comparison to women on functional reach test, the timed up and go test, the sit to stand test, and the step test in participants aged 50 to 75 years old [10]. And a study based on Danish population showed the elderly men performed worse than the elderly women in static balance [11]. A study in Finnish population elucidated effects of age, gender and their interactions on postural balance with various tests, confirmed properties of age and gender dependency [12]. Thus it could be concluded these conflicting findings could be due to contributions of ethnicities, population features, ages, measurement approaches and others.

The present results demonstrated individual variations in static balance, postural stability, dynamic balance, and overall balance performance all increased gradually and substantially over ages in the elderly. Balance performance requires profound integration of vision, vestibular sense, proprioception, muscle strength, neuromuscular, and skeletal function. With increased age, progressive dysfunctions on these systems would result in balance deficits. Where diversities in these functional disorders over age and cumulative impacts of these health inequities across life course would contribute to larger varieties in balance performance [23, 24]. Previous study showed variability in gait velocity increased in older adults compared to young and middle-aged adults, which is in line with the present findings of increasing variations across ages [25].

It was not a surprise that portions with success performance on specific tasks declined gradually with increases in age. However, there were differences in deterioration extent between tasks. In static balance, portions with success performance on 3 tasks of standing on two legs were comparable. Whereas relative to these 3 tasks, portions with success performance on standing on one leg (item 4) was substantially lower across all age groups, these obvious worsening abilities of standing on one leg hinted significantly reduced balance of dominant leg at functional level started from 60 years old and earlier.

Amongst the 4 tasks of postural stability, portions with success performance on postural transfers between standing and squatting were lower than transfers between standing and sitting, and portions with intact abilities of transferring from squatting to standing (item 8) were the lowest. Achievements of these postural transfers depend largely on strengths of lower body extremities which were reduced gradually with advances in age. The worsening severity of balance performance on specific task could be attributable to extents of dependencies on and deterioration of strengths of lower body extremities [26].

Among the 8 tasks of dynamic balance, portions of the elderly with normal step length (item 11) was the lowest in the elderly. Previous studies had revealed significant decreases in step length over ages in the elderly. The elderly tended to develop a more cautious gait which was characterized by a reduced step length consequently a reduced gait speed [27].

Collectively, the performance on standing on one leg and normal step length decreased distinctively since 70 years old, and transfers between squatting and standing postures decreased obviously as early as 65 years old, suggesting preventive and therapeutic measures aiming to improve these abilities are in urgent needs.

Given desirable features of handling categorical and continuous variables and automatic selection of number of clusters, the two-step cluster analysis was applied in this research to identify group segmentations.

The present study was based on community setting population who are functionally independent, thus the findings may not be generalized to populations with requirements for nursing or hospitalization. The X16 scale was designated for screening for high risk subpopulation from community setting or large scale study, selected individuals subject to further examination and diagnosis for subsequent treatment and intervention.

Theoretically, it was possible that the Hawthorne effect [28] might exist in the study, making the observed balance performance from the participants might be slightly better than the realities in the population.

The previous results demonstrated that both the reliability and validity of the X16 scale were adequate and acceptable [17], and difficulties of the task were taken into consideration for the level rating. However, due to practical difficulties the weights of each item have not been evaluated more exactly. The calculation of the total balance score may subject to optimization upon availability of appropriate weighting solution.

## Conclusions

In the elderly, with advances in age, abilities of overall balance performance, individual domains of static balance, postural stability, and dynamic balance, and successful performances on specific tasks declined gradually and stably. The deterioration started to be obvious since 65–75 years. Performances on standing on one leg, postural transfering from squatting to standing, and step length was worse than remaining tasks in their corresponding domains of static balance, postural stability, and dynamic balance, respectively. Men and women had similar patterns.

## Supplementary Information

**Additional file 1: Table S1** The 16 items of the X16 balance testing scale for the elderly. **Table S2** Age and gender compositions of the elderly. **Table S3** Static balance in the elderly by age and gender. **Table S4** Postural stability in the elderly men by age. **Table S5** Postural stability in the elderly women by age. **Table S6** Dynamic balance in the elderly men by age. **Table S7** Dynamic balance in the elderly women by age. **Table S8** Categories of balance performance in the elderly by age and gender.

## Data Availability

The data used in the study are available from the corresponding author on reasonable request.
