# Supplementary material for: Characteristics of balance performance in the Chinese elderly by age and gender
Source: BMC Geriatr. 2021 Oct 25;21:596. doi: 10.1186/s12877-021-02560-9 (PMC8543793; doi:10.1186/s12877-021-02560-9)
Supplement: Supplementary file 1 — Additional file 1: Table S1 The 16 items of the X16 balance testing scale for the elderly. Table S2 Age and gender compositions of the elderly. Table S3 Static balance in the elderly by age and gender. Table S4 Postural stability in the elderly men by age. Table S5 Postural stability in the elderly women by age. Table S6 Dynamic balance in the elderly men by age. Table S7 Dynamic balance in the elderly women by age. Table S8 Categories of balance performance in the elderly by age and gender. [file 12877_2021_2560_MOESM1_ESM.pdf]

Table S1 The 16 items of the X16 balance testing scale for the elderly

Domain	Item	Instruction for scoring
**I: Static balance**		
	**I 1**	**Standing with feet together**
		1: Hold the posture for more than 10 seconds
		0: Hold the posture for less than 10 seconds
	**I 2**	**Standing with one foot in front**
		1: Hold the posture for more than 10 seconds
		0: Hold the posture for less than 10 seconds
	**I 3**	**Standing with eyes closed**
		1: Hold the posture for more than 10 seconds
		0: Hold the posture for less than 10 seconds
	**I 4**	**Standing on one leg**
		1: Hold the posture for more than 10 seconds
		0: Hold the posture for less than 10 seconds
**II: Postural stability**		
	**II 5**	**Standing to sitting**
		2: Sit down easily without holding the armrest
		1: Sit down with holding the armrest or after attempts
		0: Unable to sit down without assist of others
	**II 6**	**Sitting to standing**
		2: Stand up easily without holding the armrest
		1: Stand up with holding the armrest or after attempts
		0: Unable to stand up without assist of others
	**II 7**	**Standing to squatting**
		2: Squat easily without holding the armrest
		1: Squat with holding the armrest or after attempts
		0: Unable to squat without assist of others
	**II 8**	**Squatting to standing**
		2: Stand up easily without holding the armrest
		1: Stand up with holding the armrest or after attempts
		0: Unable to stand up without assist of others
**III: Dynamic balance**		
	**III 9**	**Initiation of gait**
		1: Initiate immediately without hesitates or attempts
		0: Initiate after hesitates or attempts
	**III 10**	**Step height**
		1: Walk with the foot is lift clearly off the ground
		0: Walk with at least one foot is unable to be lift clearly off the ground
	**III 11**	**Step length**
		1: Step length longer than foot length
		0: Step length shorter than foot length
	**III 12**	**Step symmetry**
		1: Uniform step length and step height
		0: Non-uniform step length or step height
	**III 13**	**Step continuity**
		1: Walk continually

	0: Walk with stops between steps
**III 14**	**Walk path**
	1: Walk along the line
	0: Walk with deviation from the line
**III 15**	**Trunk stability while walking**
	1: Walk steadily without trunk sway or arm extension for balance
	0: Walk with trunk sway or arm extension for balance
**III 16**	**Turning while walking**
	1: Turn steadily without sway or stop
	0: Turn with sway or stop

From Ju, BMC Geriatrics, 2018 [17].

Table S2 Age and gender compositions of the elderly

Age (yrs)	n	Men		Women		Sex Ratio
		n	%	n	%	
60−	511	228	24.3	283	27.1	0.81
65−	530	246	26.2	284	27.2	0.87
70−	378	184	19.6	194	18.6	0.95
75−	297	159	16.9	138	13.2	1.15
80−	165	82	8.7	83	8.0	0.99
85−97	103	41	4.4	62	5.9	0.66
Total	1984	940	100.0	1044	100.0	0.90

Sex ratios were expressed as proportions of men to women.

Table S3 Static balance in the elderly by age and gender

Age (yrs)	Men									Women								
	n	I 1		I 2		I 3		I 4		n	I 1		I 2		I 3		I 4	
		n	%	n	%	n	%	n	%		n	%	n	%	n	%	n	%
60−	228	219	96.1^{a}	220	96.5^{a}	217	95.2^{a}	207	90.8^{a}	283	271	95.8$^{a}_{A}$	272	96.1$^{a}_{A}$	270	95.4$^{a}_{A}$	247	87.3$^{a}_{B}$
65−	246	234	95.1$^{a}_{A}$	233	94.7$^{ab}_{A}$	232	94.3$^{a}_{A}$	209	85.0$^{ab}_{B}$	284	268	94.4$^{a}_{A}$	267	94.0$^{ab}_{A}$	266	93.7$^{ab}_{A}$	237	83.5$^{ab}_{B}$
70−	184	175	95.1$^{ab}_{A}$	177	96.2$^{ab}_{A}$	173	94.0$^{ab}_{A}$	144	78.3$^{bc}_{B}$	194	177	91.2$^{ab}_{A}$	179	92.3$^{abc}_{A}$	169	87.1$^{bc}_{A}$	143	73.7$^{bc}_{B}$
75−	159	142	89.3$^{abc}_{A}$	140	88.1$^{bc}_{A}$	135	84.9$^{bc}_{A}$	102	64.2$^{cd}_{B}$	138	124	89.9$^{ab}_{A}$	121	87.7$^{bcd}_{A}$	113	81.9$^{c}_{A}$	85	61.6$^{cd}_{B}$
80−	82	70	85.4$^{bc}_{A}$	64	78.0$^{c}_{AB}$	60	73.2$^{cd}_{AB}$	50	61.0$^{cd}_{B}$	83	68	81.9$^{bc}_{A}$	68	81.9$^{cd}_{A}$	61	73.5$^{cd}_{A}$	42	50.6$^{de}_{B}$
85−97	41	33	80.5$^{c}_{A}$	30	73.2$^{c}_{A}$	25	61.0$^{d}_{AB}$	16	39.0$^{d}_{B}$	62	45	72.6$^{c}_{A}$	44	71.0$^{d}_{A}$	37	59.7$^{d}_{A}$	21	33.9$^{e}_{B}$
Total	940	873	92.9$_{A}$	864	91.9$_{A}$	842	89.6$_{A}$	728	77.4$_{B}$	1044	953	91.3$_{A}$	951	91.1$_{AB}$	916	87.7$_{B}$	775	74.2$_{C}$
χ^2		26.3		57.4		80.7		94.7			47.3		53.6		90.2		126.4	
P		<0.0001		<0.0001		<0.0001		<0.0001			<0.0001		<0.0001		<0.0001		<0.0001	

Superscript lowercase letters (a, b, c, etc) indicated multiple comparison results among various age groups. Subscript capital letters (A, B, C, etc) indicated multiple comparison results between items. Same letters indicated non-significant difference, different letters indicated significant differences in statistics. Significance level was 0.05.

Table S4 Postural stability in the elderly men by age

Age (yrs)	n	II 5						II 6						II 7						II 8					
		2		1		0		2		1		0		2		1		0		2		1		0	
		n	%	n	%	n	%	n	%	n	%	n	%	n	%	n	%	n	%	n	%	n	%	n	%
60−	228	224	98.2^{a}_{A}	4	1.8	0	0.0	220	96.5^{a}_{A}	8	3.5	0	0.0	215	94.3^{a}_{AB}	12	5.3	1	0.4	204	89.5^{a}_{B}	23	10.1	1	0.4
65−	246	225	91.5^{b}_{A}	20	8.1	1	0.4	223	90.7^{a}_{AB}	22	8.9	1	0.4	211	85.8^{b}_{AB}	33	13.4	2	0.8	204	82.9^{ab}_{B}	39	15.9	3	1.2
70−	184	171	92.9^{ab}_{A}	13	7.1	0	0.0	172	93.5^{a}_{A}	11	6.0	1	0.5	154	83.7^{b}_{B}	30	16.3	0	0.0	143	77.7^{b}_{B}	39	21.2	2	1.1
75−	159	126	79.2^{c}_{A}	32	20.1	1	0.6	117	73.6^{b}_{AB}	40	25.2	2	1.3	99	62.3^{c}_{BC}	53	33.3	7	4.4	93	58.5^{c}_{C}	59	37.1	7	4.4
80−	82	55	67.1^{cd}_{AB}	25	30.5	2	2.4	56	68.3^{bc}_{B}	25	30.5	1	1.2	39	47.6^{c}_{A}	37	45.1	6	7.3	41	50.0^{c}_{AB}	33	40.2	8	9.8
85−97	41	20	48.8^{d}	19	46.3	2	4.9	17	41.5^{c}	22	53.7	2	4.9	16	39.0^{c}	21	51.2	4	9.8	14	34.1^{c}	20	48.8	7	17.1
Total	940	821	87.3_{A}	113	12.0	6	0.6	805	85.6_{A}	128	13.6	7	0.7	734	78.1_{B}	186	19.8	20	2.1	699	74.4_{B}	213	22.7	28	3.0
χ^2		128.5						139.9						151.4						119.2					
P		<0.0001						<0.0001						<0.0001						<0.0001					

Superscript lowercase letters (a, b, c, etc) indicated multiple comparison results among various age groups. Subscript capital letters (A, B, C, etc) indicated multiple comparison results between items. Same letters indicated non-significant difference, different letters indicated significant differences in statistics. Significance level was 0.05.

Table S5 Postural stability in the elderly women by age

Age (yrs)	n	II 5						II 6						II 7						II 8					
		2		1		0		2		1		0		2		1		0		2		1		0	
		n	%	n	%	n	%	n	%	n	%	n	%	n	%	n	%	n	%	n	%	n	%	n	%
60−	283	268	94.7$^{a}_{A}$	15	5.3	0	0.0	263	92.9$^{a}_{AB}$	20	7.1	0	0.0	253	89.4$^{a}_{AB}$	30	10.6	0	0.0	246	86.9$^{a}_{B}$	37	13.1	0	0.0
65−	284	267	94.0$^{a}_{A}$	16	5.6	1	0.4	257	90.5$^{a}_{A}$	26	9.2	1	0.4	226	79.6$^{b}_{B}$	55	19.4	3	1.1	215	75.7$^{b}_{B}$	65	22.9	4	1.4
70−	194	179	92.3$^{a}_{A}$	13	6.7	2	1.0	170	87.6$^{ab}_{A}$	23	11.9	1	0.5	139	71.6$^{bc}_{B}$	50	25.8	5	2.6	131	67.5$^{bc}_{B}$	54	27.8	9	4.6
75−	138	108	78.3$^{b}_{A}$	28	20.3	2	1.4	105	76.1$^{bc}_{AB}$	31	22.5	2	1.4	90	65.2$^{c}_{AB}$	43	31.2	5	3.6	87	63.0$^{bc}_{B}$	44	31.9	7	5.1
80−	83	53	63.9^{b}	29	34.9	1	1.2	54	65.1^{cd}	28	33.7	1	1.2	45	54.2^{cd}	37	44.6	1	1.2	44	53.0^{cd}	38	45.8	1	1.2
85−97	62	31	50.0^{c}	24	38.7	7	11.3	27	43.5^{d}	27	43.5	8	12.9	23	37.1^{d}	26	41.9	13	21.0	22	35.5^{d}	27	43.5	13	21.0
Total	1044	906	86.8$_{A}$	125	12.0	13	1.2	876	83.9$_{A}$	155	14.8	13	1.2	776	74.3$_{B}$	241	23.1	27	2.6	745	71.4$_{B}$	265	25.4	34	3.3
χ^2		153.4						131.1						107.2						95.0					
P		<0.0001						<0.0001						<0.0001						<0.0001					

Superscript lowercase letters (a, b, c, etc) indicated multiple comparison results among various age groups. Subscript capital letters (A, B, C, etc) indicated multiple comparison results between items. Same letters indicated non-significant difference, different letters indicated significant differences in statistics. Significance level was 0.05.

Table S6 Dynamic balance in the elderly men by age

Age (yrs)	n	III 9		III 10		III 11		III 12		III 13		III 14		III 15		III 16	
		n	%	n	%	n	%	n	%	n	%	n	%	n	%	n	%
60−	228	223	97.8^{a}	220	96.5^{a}	217	95.2^{a}	222	97.4^{a}	222	97.4^{a}	224	98.2^{a}	224	98.2^{a}	222	97.4^{a}
65−	246	233	94.7^{a}	226	91.9^{ab}	222	90.2^{ab}	234	95.1^{a}	232	94.3^{a}	235	95.5^{ab}	230	93.5^{ab}	234	95.1^{a}
70−	184	175	95.1^{a}	171	92.9^{ab}	162	88.0^{ab}	171	92.9^{ab}	174	94.6^{a}	176	95.7^{ab}	176	95.7^{ab}	170	92.4^{ab}
75−	159	135	84.9^{b}	132	83.0^{bc}	129	81.1^{bc}	136	85.5^{bc}	131	82.4^{b}	142	89.3^{bc}	144	90.6^{bc}	134	84.3^{bc}
80−	82	66	80.5^{b}	63	76.8^{cd}	53	64.6^{cd}	61	74.4^{c}	60	73.2^{bc}	68	82.9^{c}	67	81.7^{c}	62	75.6^{c}
85−97	41	30	73.2^{b}	25	61.0^{d}	19	46.3^{d}	28	68.3^{c}	23	56.1^{c}	32	78.0^{c}	31	75.6^{c}	28	68.3^{c}
Total	940	862	91.7_{AB}	837	89.0_{BC}	802	85.3_{C}	852	90.6_{AB}	842	89.6_{ABC}	877	93.3_{A}	872	92.8_{AB}	850	90.4_{AB}
χ^2		58.6		69.4		103.5		73.7		107.3		45.9		46.8		70.7	
P		<0.0001		<0.0001		<0.0001		<0.0001		<0.0001		<0.0001		<0.0001		<0.0001	

Superscript lowercase letters (a, b, c, etc) indicated multiple comparison results among various age groups. Subscript capital letters (A, B, C, etc) indicated multiple comparison results between items. Same letters indicated non-significant difference, different letters indicated significant differences in statistics. Significance level was 0.05.

Table S7 Dynamic balance in the elderly women by age

Age (yrs)	n	III 9		III 10		III 11		III 12		III 13		III 14		III 15		III 16	
		n	%	n	%	n	%	n	%	n	%	n	%	n	%	n	%
60−	283	275	97.2^{a}_{A}	274	96.8^{a}_{A}	253	89.4^{a}_{B}	273	96.5^{a}_{A}	274	96.8^{a}_{A}	278	98.2^{a}_{A}	277	97.9^{a}_{A}	274	96.8^{a}_{A}
65−	284	273	96.1^{ab}_{A}	266	93.7^{a}_{AB}	246	86.6^{ab}_{B}	268	94.4^{ab}_{A}	269	94.7^{a}_{A}	278	97.9^{a}_{A}	277	97.5^{a}_{A}	273	96.1^{a}_{A}
70−	194	183	94.3^{ab}_{AB}	178	91.8^{ab}_{AB}	169	87.1^{ab}_{B}	179	92.3^{ab}_{AB}	179	92.3^{ab}_{AB}	190	97.9^{a}_{A}	188	96.9^{ab}_{A}	178	91.8^{ab}_{AB}
75−	138	123	89.1^{bc}_{AB}	117	84.8^{bc}_{AB}	108	78.3^{bc}_{B}	121	87.7^{bc}_{AB}	113	81.9^{bc}_{AB}	128	92.8^{ab}_{A}	125	90.6^{bc}_{AB}	117	84.8^{bc}_{AB}
80−	83	64	77.1^{cd}	63	75.9^{cd}	55	66.3^{cd}	65	78.3^{cd}	60	72.3^{cd}	71	85.5^{bc}	65	78.3^{cd}	62	74.7^{cd}
85−97	62	37	59.7^{d}	34	54.8^{d}	32	51.6^{d}	36	58.1^{d}	35	56.5^{d}	43	69.4^{c}	40	64.5^{d}	33	53.2^{d}
Total	1044	955	91.5_{ABC}	932	89.3_{BC}	863	82.7_{D}	942	90.2_{BC}	930	89.1_{C}	988	94.6_{A}	972	93.1_{AB}	937	89.8_{BC}
χ^2		125.0		118.9		73.9		106.1		128.0		109.8		131.7		142.8	
P		<0.0001		<0.0001		<0.0001		<0.0001		<0.0001		<0.0001		<0.0001		<0.0001	

Superscript lowercase letters (a, b, c, etc) indicated multiple comparison results among various age groups. Subscript capital letters (A, B, C, etc) indicated multiple comparison results between items. Same letters indicated non-significant difference, different letters indicated significant differences in statistics. Significance level was 0.05.

Table S8 Categories of balance performance in the elderly by age and gender

Age (yrs)	Men							Women						
	n	Poor (0−10)		Fair (11−17)		Good (18−20)		n	Poor (0−10)		Fair (11−17)		Good (18−20)	
		n	%	n	%	n	%		n	%	n	%	n	%
60−	228	2	0.9[a]	19	8.3[a]	207	90.8[a]	283	6	2.1[a]	23	8.1[a]	254	89.8[a]
65−	246	7	2.8[a]	36	14.6[ab]	203	82.5[a]	284	6	2.1[a]	48	16.9[b]	230	81.0[b]
70−	184	8	4.3[ab]	24	13.0[ab]	152	82.6[a]	194	10	5.2[a]	42	21.6[bc]	142	73.2[bc]
75−	159	20	12.6[bc]	39	24.5[bc]	100	62.9[b]	138	11	8.0[ab]	43	31.2[c]	84	60.9[cd]
80−	82	19	23.2[c]	22	26.8[bc]	41	50.0[bc]	83	18	21.7[bc]	25	30.1[bc]	40	48.2[de]
85−97	41	12	29.3[c]	19	46.3[c]	10	24.4[c]	62	26	41.9[c]	15	24.2[bc]	21	33.9[e]
Total	940	68	7.2	159	16.9	713	75.9	1044	77	7.4	196	18.8	771	73.9

A two-step cluster analysis was applied to identify group segmentations. Superscript lowercase letters (a, b, c, etc) indicated multiple comparison results among various age groups. Same letters indicated non-significant difference, different letters indicated significant differences in statistics. Significance level was 0.05.
